# Caveolin-1 and MLRs: A potential target for neuronal growth and neuroplasticity after ischemic stroke

**DOI:** 10.7150/ijms.35158

**Published:** 2019-10-15

**Authors:** Wei Zhong, Qianyi Huang, Liuwang Zeng, Zhiping Hu, Xiangqi Tang

**Affiliations:** Department of Neurology, The Second Xiangya Hospital, Central South University, Changsha, Hunan 410011, China

**Keywords:** Caveolin-1, membrane lipid raft, ischemic stroke, neuronal growth, neuroplasticity, non-coding RNA.

## Abstract

Ischemic stroke is a leading cause of morbidity and mortality worldwide. Thrombolytic therapy, the only established treatment to reduce the neurological deficits caused by ischemic stroke, is limited by time window and potential complications. Therefore, it is necessary to develop new therapeutic strategies to improve neuronal growth and neurological function following ischemic stroke. Membrane lipid rafts (MLRs) are crucial structures for neuron survival and growth signaling pathways. Caveolin-1 (Cav-1), the main scaffold protein present in MLRs, targets many neural growth proteins and promotes growth of neurons and dendrites. Targeting Cav-1 may be a promising therapeutic strategy to enhance neuroplasticity after cerebral ischemia. This review addresses the role of Cav-1 and MLRs in neuronal growth after ischemic stroke, with an emphasis on the mechanisms by which Cav-1/MLRs modulate neuroplasticity via related receptors, signaling pathways, and gene expression. We further discuss how Cav-1/MLRs may be exploited as a potential therapeutic target to restore neuroplasticity after ischemic stroke. Finally, several representative pharmacological agents known to enhance neuroplasticity are discussed in this review.

## 1. Introduction

Ischemic stroke is a common nervous system disease associated with high rates of disability and mortality. Ischemic stroke results from disruption of blood supply, resulting in hypoxic necrosis of brain tissue, and manifestation of corresponding neurological deficits 1. The most effective treatment for acute ischemic stroke is intravenous administration of recombinant tissue plasminogen activator (rt-PA) within 3-4.5 hours after stroke to induce thrombolysis. However, less than 5% of patients are able to receive thrombolytic therapy within the critical time window because they do not meet the criteria for thrombolysis. In addition, owing to increased risk of hemorrhagic transformation, clinical application of thrombolytic therapy is limited 2. There is currently no therapeutic strategy to improve stroke-related deficits. Recent studies have focused on identification of effective neuroprotectants and nerve repair drugs to protect brain tissue and promote neuronal growth and neuroplasticity following ischemic stroke.

This review will highlight the role of Cav-1 and MLRs in neuronal growth following ischemic stroke, with an emphasis on the mechanisms by which Cav-1/MLRs modulate neuroplasticity via related receptors, signaling pathways, and genes. Potential clinical applications will also be discussed.

## 2. Cav-1 and MLRs in neuronal growth and neuroplasticity after ischemic stroke

Recent studies have shown that new neurons are born during cerebral ischemia, and the underlying mechanisms of neuroplasticity may provide a basis for pharmacological enhancement of treatment of ischemic stroke 3. Neuroplasticity includes structural and functional plasticity 4. Structural plasticity is characterized by changes in neurite length, dendritic spine density, and synapse number. Functional plasticity is characterized by changes in synaptic transmission efficiency 5. Neuroplasticity involves angiogenesis, nerve regeneration, and synaptogenesis 6, which includes proliferation, migration, and differentiation of neural stem cells (NSCs) to mature neurons 7. Neurotrophins (NTs) such as nerve growth factor (NGF), brain derived neurotrophic factor (BDNF), and neurotrophic factors NT3, NT4 and NT5 are important promoters of neuroplasticity 8. Neurotrophins exert physiological effects through specific binding to receptors on cell membranes (including Trk-A, Trk-B, Trk-C, etc.). Neurotrophin binding to receptors occurs in discontinuous regions of neuronal cell membranes called membrane lipid rafts (MLR), which are crucial structures for neuronal survival and function of growth signaling pathways 9. Furthermore, MLRs are crucial to development, stability, and maintenance of synapses 10.

MLRs are rich in cholesterol, sphingomyelin, and scaffold proteins. In addition, caveolin, a scaffolding and cholesterol-binding protein, is enriched in MLRs. Caveolin is a structural component of caveolae, which are components of lipid rafts, and are highly ordered microdomains located in the plasma membrane 11. The caveolin family has three members in mammals: caveolin-1 (Cav-1), caveolin-2 (Cav-2), and caveolin-3 (Cav-3). Cav-1 is expressed ubiquitously, but at different levels in different tissues. Caveolin-2 is co-expressed with Cav-1, and Cav-3 is expressed predominantly in muscle cells, such as skeletal, smooth, and cardiac myocytes 12. In the brain, Cav-1 and Cav-2 are primarily expressed in endothelial cells and neurons, and Cav-3 is expressed in astrocytes 13. Caveolin-2 may not be essential for caveolae formation, as caveolae formation is not affected in Cav-2-knockout mice 14.

Neurite and dendrite outgrowth consists of protrusion, engorgement, and consolidation. Membrane lipid rafts are located at the leading edge of neuronal growth cones, providing an essential plasma membrane platform to establish cellular polarity and to compartmentalize pro-growth signaling components 15. Caveolin-1 is the main cholesterol binding protein in MLRs, and is important in many cellular functions 16. In addition, Cav-1 is a target protein for many neuronal growth-promoting proteins expressed in MLRs, which promote growth of neurons and dendrites 17. In addition, Cav-1 is a regulator of membrane cholesterol, and is directly involved in synthesis and transport of intracellular cholesterol, which is important for maintenance of cholesterol homeostasis and formation of MLRs 18. A recent study showed that Cav-1 regulates N-cadherin and L1CAM trafficking independent of caveolae, resulting in immature neurite pruning and early neuronal maturation 19. Many neurodegeneration studies have shown that neuron-targeted overexpression of Cav-1 increased MLR formation, pro-growth receptor localization to MLRs, myelination, and long-term potentiation, resulting in neuronal development and regeneration 20-23. Furthermore, synapsin-driven overexpression of Cav-1 was shown to preserve and restore NT-receptors expression and localization to MLRs, resulting in delayed progression of ALS in a mouse model 24. These studies suggested that Cav-1 and MLRs may be potential therapeutic targets to promote neuroplasticity in neurological disorders. Caveolin-1 may be a key factor in maintenance of MLRs and neuroplasticity after ischemic stroke.

## 3. Role of receptors associated with Cav-1 and MLRs in neuronal growth and neuroplasticity after ischemic stroke

Following ischemic stroke, disruption of pro-survival and pro-growth signaling pathways limits neuroplasticity and subsequent recovery. Several receptors associated with Cav-1 and MLRs are crucial to neuroplasticity, and may be potential therapeutic targets to improve functional recovery after ischemic stroke.

### 3.1. Src family kinases

The Src family kinases (SFKs) are non-receptor tyrosine kinases involved in signal transduction that modulates cell morphology, adhesion, migration, invasion, proliferation, differentiation, and survival 25. Caveolin was discovered as a phosphorylation target of the kinase encoded by Rous sarcoma virus, v-Src kinase, which was the first tyrosine kinase to be identified 26. Phosphorylation of caveolin at Tyr14 (pY14-Cav1) inhibits Src through recruitment of C-terminal Src kinase 27. In mouse embryonic fibroblasts (MEFs), Cav-1 knockout increased the activation of Src, resulting in morphological changes and inhibition or polarization and directed motility 28. In addition, aggregation of Cav-1 and MLR has been show to activate the proto-oncogene tyrosine protein kinase Src (c-Src) to induce gastric cancer cell migration 29. Src has also been shown to modulate neuronal growth and oligodendrocyte maturation 30. Another study showed that Cav-1 activated Src and enhanced N-methyl-D-aspartate receptor (NMDAR) localization on MLRs, resulting in protection against hypoxia in cultured neonatal rat neurons 31. Based on these findings, modulation of Src and Cav-1 may provide a novel approach to promote neuronal growth after ischemic stroke.

### 3.2. Tropomyosin-related kinase receptors

Neurotrophins are a family of growth factors that mediate development and survival of neurons and glial cells. Furthermore, NTs are essential effectors of neuroplasticity 32. Many signals elicited by neurotrophins (NGF, BDNF, and NT3,4) require binding to the tropomyosin-related kinase (Trk) receptor family (TrkA, TrkB, and TrkC) to activate downstream signaling pathways 33, and the combination process was performed in MLRs 21. Limitation of functional recovery following stroke is primarily a consequence of downregulation of pro-growth and pro-survival signaling through pathways such as the TrkB signaling pathway 34. Therefore, interventions that upregulate pro-growth and pro-survival signaling pathways may improve functional outcomes. A previous study showed that Cav-1 may exert protective effects against ischemia/reperfusion injury 35. For example, neuron-targeted Cav-1 (Syn-Cav1) overexpression concentrated TrkB receptors in MLRs, resulting in enhanced dendritic growth and arborization of primary neurons in mice 22. Conversely, another study showed that overexpression of Cav-1 in PC12 cells blocked NGF-mediated TrkA autophosphorylation, resulting in inhibition of neurite outgrowth 36. Thus, Cav-1 may act through TrkB to promote nerve regeneration after ischemic stroke.

### 3.3. N-methyl-D-aspartate receptors

N-methyl-D-aspartate (NMDA) receptors, alpha-amino-3-hydroxy-5-methyl-4-isoxazolepropionic acid (AMPA) receptors, and kainate receptors are the three classes of ionotropic glutamate receptors (iGluRs). These receptors are critical to neuronal development and synaptic plasticity 37. NMDA receptors located in MLRs as a component of the neurotransmitter and neurotrophic receptors 22. Cav-1 has been shown to regulate neuroplasticity and long-term plastic changes related to NMDAR2B, resulting in modulation of chronic pain 38. Furthermore, Cav-1 overexpression has been shown to enhance MLR formation and enrichment of NMDAR2B in MLRs, resulting in improved motor function and preservation of memory in mice subjected to brain trauma 23. In addition, a study showed that siRNA-mediated knockdown of Cav-1 disrupted NMDA2A-mediated signaling and attenuated neuroprotection following oxygen and glucose deprivation 31. These findings demonstrate the potential beneficial effects of Cav-1/NMDAR on neuroplasticity.

### 3.4. G-protein coupled receptors

G-protein coupled receptors (GPCRs) are a large family of transmembrane signaling receptors that bind to extracellular molecules to produce intracellular signals 39. Pro-growth signaling in neurons has been shown to occur following activation of a number of synaptic receptors, including GPCRs 40. Studies have shown a close relationship between GPCRs and caveolin, with caveolin-rich domains organized proximal to GPCR signaling components in both the sarcolemmal and intracellular regions in rat heart 41. Inactive Gα subunits are concentrated in caveolae and associate with the caveolin scaffolding domain (CSD). Upon activation, Gα subunits dissociate from caveolae 42. Caveolin-1 and MLRs have been shown to modulate estrogen GPCR signaling in the nervous system 43. Interestingly, Cav-1-mediated NMDA receptor activation may be coordinated by the Gαq subunit, resulting in modulation of pro-growth pathways in oxygen-glucose-deprived (OGD) neurons 31. These findings suggest an important role for GPCRs alone or in combination with other receptors in Cav-1-mediated neuroplasticity.

### 3.5. Other receptors related to neuronal growth and neuroplasticity

Modulation of neuroplasticity by Cav-1 may depend on several mechanisms, including angiogenesis. The role of vascular endothelial growth factor (VEGF) in angiogenesis following ischemia has been established 44. Caveolin-1 has been shown to colocalize with VEGF receptor 2 (VEGFR2), resulting in increased VEFGR2 autophosphorylation and activation of downstream angiogenic signaling in prostate cancer and in endothelial cells 45. Studies have shown that treadmill exercise promoted NSC proliferation, migration, and neuronal differentiation, and improved neurological recovery via Cav-1/VEGF signaling after ischemic injury 46, 47. In addition, fibroblast growth factor (FGF) has also been shown to exert protective effects against brain ischemia. Caveolin-1 was shown to interact with FGF receptor 1 (FGFR1) to regulate FGF-2-induced angiogenesis in ovine placental artery endothelial cells 48.

Cellular prion protein (PrP^c^), a ubiquitous glycoprotein expressed strongly in neurons, acts as a cell-surface receptor and plays an important role in regulation of neuronal differentiation and neurite growth 49. MLRs are critical to conformational changes in PrP^c^ that promote signal transduction and neurite outgrowth 50. Pantera et al. demonstrated that PrP^c^-mediated neuritogenesis and cell differentiation occurred through increased phosphorylation of Cav-1 in PC12 cells 51. Bone morphogenetic proteins (BMPs), members of the growth factor β (TGF β) family, are important in osteogenesis and neuroplasticity. The BMP receptors BRIa and BRII have been shown to colocalize with Cav-1 52. Colocalization of BMPRII with Cav-1 has been shown to regulate downstream signaling in vascular smooth muscle cells 53, resulting in stem cell differentiation 54. The prorenin receptor ATP6AP2 may also affect neuroplasticity. Gα proteins can crosslink ATP6AP2 to caveolin where a switch from Gαi to Gαq was necessary to induce neuronal differentiation of adipose-derived mesenchymal stem cells (MSCs) 55. Caveolin-1 is necessary for glucocorticoid receptor (GRs)-mediated proliferation of neural progenitor cells (NPCs) 56.

## 4. Signaling pathways mediated by Cav-1 and MLRs in neuronal growth and neuroplasticity after ischemic stroke

Several classical signaling pathways participate in neuronal growth and neuroplasticity. Caveolin-1 has been shown to directly or indirectly regulate signaling by concentrating signal transducers within distinct membrane regions 57. Recent studies have focused on increasing understanding of intracellular signaling pathways linked to neuroplasticity. Elucidation of signaling mechanisms involving Cav-1 will aid in identification of new therapeutic targets.

### 4.1. PI3k/Akt signaling pathway

The phosphatidylinositol-3-kinase (PI3K) pathway is a well-characterized pathway that regulates endogenous neuroplasticity in response to ischemia 58. Studies have shown that activation of the PI3K pathway promotes brain cell survival, resulting in reduced cell death after stroke 59, 60. PI3K activates many different downstream effectors, such as Akt (via phosphorylation), which promotes growth, translation, and cell-cycle regulation 61. Caveolin-1 has been shown to interact with the PI3k/Akt pathway. For example, previous studies showed that Cav-1 enhanced PI3k/Akt signaling, resulting in human MSC osteogenesis 62, alleviated the effects of ischemia-reperfusion injury in the diabetic myocardium 63, and increased morphine-induced neuroplasticity 57. Another study showed that angiotensin II-induced remodeling of cerebral pial arterioles occurred via the Cav-1/Akt pathway 64. Caveolin-1 overexpression has been shown to augment phosphorylation of Akt and to enhance dendritic growth in response to ischemic injury 31. In contrast, other studies have shown that endothelial cell-specific expression of Cav-1 inhibited the Akt-endothelial nitric oxide synthase (eNOS) pathway and impaired microvascular angiogenesis 65, and Cav-1 decreased Akt and Stat3 phosphorylation, resulting in inhibition of neuronal differentiation of NPCs 66. Therefore, determination of whether Cav-1 inhibits or activates the PI3k/Akt pathway requires further investigation.

### 4.2. MAPK/ERK signaling pathway

Extracellular signal-regulated kinase (ERK) is a member of the mitogen-activated protein kinase (MAPK) family, which transduces signals from the cell membrane to the nucleus to promote cell proliferation, migration, differentiation, and death 67. A previous report suggested that Cav-1 can act as a negative regulator of the Ras-p42/44 ERK pathway in a variety of cell types 68. Furthermore, Cav-1 has been shown to downregulate matrix metalloproteinase-1(MMP-1) expression via inhibition of ERK1/2/Ets1 signaling 69. In contrast, a study showed that insulin-activated ERK translocation to the nuclear envelope was Cav-2-dependent 70. Glial cell line-derived neurotrophic factor (GDNF) stimulation induced upregulation of caveolin and ERK expression in neurons, resulting in increased ERK signaling. GDNF-induced increases in ERK signaling were blocked by inhibition of caveolin 71. In primary astrocytes exposed to OGD, overexpression of Cav-1 and increased phospho-ERK attenuated OGD-induced cell apoptosis 72. Caveolin-1 expression was shown to be critical for nitric oxide-mediated angiogenesis through augmentation of ERK phosphorylation 73. Furthermore, Cav-1 overexpression enhanced dendritic growth partly due to increased ERK phosphorylation in ischemic injury 31. Li et al. showed biphasic regulation of Cav-1 gene expression through the MAPK/ERK signaling pathways following fluoxetine treatment in astrocytes 74. Further characterization of the interactions between Cav-1 and the MAPK/ERK signaling pathways is needed.

### 4.3. NF-κB signaling pathway

Nuclear factor-kappa B (NF-κB) is a transcription factor comprised of p50, RelA/p65, c-Rel, RelB, and p52 subunits that can regulate growth and elaboration of neural processes, and protect neurons against ischemia-induced neurodegeneration 75. NF-κB was shown to restore neuronal growth and differentiation through NMDAR, NGF, and NGFR signaling in hippocampus of degenerative brain 76. One study showed that NF-κB regulated dendritic spine and synapse density in the hippocampus using a p65^-/-^ model, which provided a structural basis for facilitation of learning and memory 77. Caveolin-1 can facilitate activation of NF-κB through multiple pathways. For example, β-carotene induced downregulation of Cav-1, resulting in modulation of the Akt/NF-κB pathway to induce apoptosis in human esophageal squamous cell carcinoma 78. Furthermore, Cav-1 was shown to regulate the inflammatory response through the STAT3/NF-κB pathway in mice with pseudomonas aeruginosa infection 79. In addition, Cav-1 responded to inflammation through the cPLA2/p38/NF-κB 80 and eNOS/NO/NF-κB 81 pathways. Further study is needed to determine the mechanisms by which Cav-1 promotes neuroplasticity following cerebral ischemia through the NF-κB pathway.

### 4.4. Sonic hedgehog signaling pathway

The sonic hedgehog (Shh) signaling pathway mediates neuroprotection and neuroplasticity in various types of neurons in the central nervous system 82. Sonic hedgehog signaling was shown to stimulate neurite outgrowth in astrocytes treated with cyclopamine 83. Similarly, amygdala neuronal growth was promoted by Shh signaling to form long-term memories 84 and to eliminate fear memories 85. Another study showed that Shh signaling stimulated NPC proliferation following ischemic stroke 86. The Shh pathway may have mediated this proliferative effect through cerebrolysin-enhanced neuroplasticity 87. Several treatment agents have been shown to enhance neuroplasticity via the Shh pathway following ischemia, such as salvianolic acid 88 and resveratrol 89. Studies have shown that Shh was enriched in MLRs and was activated by Cav-1 during endocytosis 90 and other Shh-related biological and pathological process 91. Another study found that Shh was associated with Cav-1 in the Golgi apparatus to form protein complexes which were transported to MLRs 92. These data suggest that Cav-1 and MLRs may be important factors in Shh signaling-induced neuroplasticity following ischemic stroke.

### 4.5. cAMP signaling pathways

Cyclic adenosine monophosphate (cAMP) signaling in the brain has been shown to mediate numerous neural processes including development, synaptic plasticity, learning and memory, and motor function in response to neurodegeneration 93. The cAMP-PKA pathway was shown to regulate synaptic plasticity in medium spiny neurons (MSNs) of the striatum 94 and to activate protein kinase A and p190B RhoGAP, resulting in neurite outgrowth in PC12 cells 95. In addition, Cav-1 may stimulate cAMP/PKA pathway-dependent lipolysis via autocrine production of PGI2 96. Caveolin-1 knockout decreased cAMP levels and PKA phosphorylation, resulting in exacerbation of cardiac dysfunction and reduced survival time of mice subjected to myocardial infarction 97. Furthermore, NMDA receptors, GPC receptors, the PI3K/Akt pathway, and the ERK pathway have been shown to enhance cAMP formation to promote neuronal growth and synaptic plasticity 98. Head et al. showed that Cav-1 stimulated cAMP formation through activation of these signaling pathways to enhance dendritic growth 22.

### 4.6. Other signaling pathways

The Notch and Wnt/β-catenin pathways have been shown to play important roles in regulation of neuroplasticity following cerebral ischemia 99. Studies have shown that Notch signaling is a pivotal control mechanisms of NSCs, and NSCs express Notch receptors and the canonical Notch target, hairy enhancer of split 5 (Hes5) 100. Notch1 signaling has been shown to modulate subventricular zone (SVZ) neuroplasticity in aged brains under normal and ischemic conditions 101. Furthermore, Caveolin-1 has been shown to promote ovarian cancer chemoresistance through Notch-1/Akt pathway-mediated inhibition of apoptosis 102. A study showed that Cav-1-containing MLRs coordinated the Notch1 and β1-integrin signaling pathways in NSCs 103. Moreover, Cav-1 has been shown to regulate neural differentiation of bone MSCs to neurons 104, and NPCs to astrocytes 105, through modulation of Notch signaling. In contrast, a study showed that Cav-1 inhibited the Wnt/β-catenin pathway, resulting in reduced dorsal organizer formation in zebrafish 106 and reduced mammary stem cell number 107. Li et al. demonstrated that Cav-1 inhibited differentiation of NSCs/NPCs into oligodendrocytes through modulation of β-catenin expression 108.

## 5. Non-coding RNAs regulate Cav-1 in neuronal growth and neuroplasticity after ischemic stroke

In addition to the classical non-coding RNAs (ncRNAs), transfer RNA (tRNA) and ribosomal RNA (rRNA), additional families of ncRNAs such as microRNAs (miRNAs), long non-coding RNAs (lncRNAs), small nuclear RNAs (snRNA), small nucleolar RNAs (snoRNA), and piwi-interacting RNAs (piRNAs) 109 have been investigated in ischemic stroke.

### 5.1. MicroRNAs

MicroRNAs (miRNAs) are a family of short non-coding RNA molecules that play important roles in gene expression via mRNA destabilization and translational repression 110. Some miRNAs have been shown to regulate normal physiological activity and response to ischemic injury 111. Recent studies have indicated that a number of miRNAs may be involved in regulation of neuroplasticity induced by cerebral ischemia 112. For example, miR-210 was upregulated in OGD PC12 cells and suppressed apoptosis by inhibiting caspase activity 113, and miR-210 overexpression increased the number of NPCs in the ischemic mouse brain 114. Furthermore, overexpression of the miR17-92 cluster enhanced stroke-induced NPC proliferation 115.

MiR-124, the most abundant microRNA in the adult brain, positively modulated differentiation of SVZ stem cells to neurons 116, a process that has been extensively investigated in neuroplasticity. MiR-124 has been shown to promote neuronal growth and neuroplasticity in various physiological processes, such as synaptic plasticity and memory formation 117. Following focal cerebral ischemia, miR-124 promoted neuronal differentiation and modulated microglia polarization 118. Interestingly, miR-124 has been linked to caveolae under many conditions. One study showed that MiR-124 regulated the expression of flotillin-2 and Cav-1 during acrosome biogenesis 119. Another study indicated that miR-124 directly bound to Cav-1 mRNA and decreased Cav-1 expression at both the mRNA and protein levels 120. Furthermore, miR-124 attenuated apoptosis by regulating the Cav-1/PI3K/Akt/GSK3β pathway in Alzheimer's disease 121, and promoted stroke-induced neuroplasticity by targeting the Notch signaling pathway 122.

The role of MiR-199 in ischemia-induced neuroplasticity has been studied extensively. A study showed that downregulation of miR-199a mediated neuroprotective effects in brain ischemic tolerance 123. Similarly, sequestration of MiR-199a by lncRNA-Map2k4 promoted FGF-1 expression and neuronal proliferation in spinal cord injury 124. Conversely, Bao et al. found that miR-199a-5p protected the spinal cord against ischemia/reperfusion-induced injury 125. Moreover, miR-199a and miR-199b were shown to modulate endocytosis by controlling the expression Cav-1, and miR-199a-5p and miR-199b-5p overexpression markedly inhibited Cav-1 expression 126. Furthermore, a study showed that miR-199a-5p downregulated Cav-1 in porcine preadipocyte proliferation and differentiation 127. Thus, miR-199a may act as a negative regulator of Cav-1 in neuroplasticity following ischemic stroke.

Studies have also indicated that other miRNAs may regulate caveolin and other signaling pathways involved in neuroplasticity. MiR-22 was reported to protect neurons against cerebral ischemia/reperfusion injury 128, and to induce protective effects against cardiac infarction through Cav-3/eNOS signaling 129. MiR-132 was shown to enhance dendritic morphogenesis, synaptic integration, and neuronal survival, and to improve outcomes of transplant therapies in olfactory bulb neurons 130. Moreover, miR-132-3p activated the PTEN/PI3K/PKB/Src/Cav-1 signaling pathway to promote transcellular transport in glioma endothelial cells 131. Transfer of MiR-133 to neural cells contributed to neurite outgrowth in rats subjected to middle cerebral artery occlusion (MCAO) 132. In contrast, MiR-133 overexpression suppressed Cav-1-mediated tumor cell proliferation, migration, and invasion 133. MiR-138 attenuated PC12 cell proliferation following hypoxia and reoxygenation 134. Downregulation of miR-138 promoted MLR formation via upregulation of Flot-1, Flot-2, and Cav-1 135. MiR-192 also suppressed cell proliferation through downregulation of Cav-1 136. Further studies to characterize the interactions between miRNAs and Cav-1 are needed.

### 5.2. Long non-coding RNAs

Long non-coding RNAs (lncRNAs) are defined as transcripts longer than 200 nucleotides without an open reading frame. More than half of lncRNAs are expressed in the central nervous system, and they play key roles in brain development and function 137. A previous study showed that of 8,314 lncRNAs analyzed, the expression levels of 443 were significantly altered at 3, 6, and 12 hours after ischemia in rats 138. Ayana et al. identified 222 lncRNAs specifically expressed in the subventricular and subgranular zones (SVZ/SGZ), and 54 of these lncRNAs were significantly up-regulated in neurogenesis zones 139. Recent studies have demonstrated that lncRNAs such as NBAT-1 140, FMR4 141, PnKy 142, Gm15577 143, and NONHSAT073641 144 play important roles in neuronal growth and neuroplasticity. Several lncRNAs have been shown to activate signaling pathways related to neuroplasticity. For example, Malat1 promoted neuronal differentiation through activation of the ERK/MAPK signalling pathway in N2a cells 145. Furthermore, LncND mediated regulation of Notch signaling to enhance NPC growth 146. Interestingly, the lncRNA HOTAIR promoted ischemic infarction through regulation of NOX2 expression in a rat model 147, and Cav-1 promoted proliferation, migration, and invasion through HOTAIR in lung cancer cells 148. Further studies to characterize interactions between Cav-1 and lncRNAs in neuroplasticity after stroke are needed.

## 6. Agents that modulate neuronal growth and neuroplasticity through Cav-1 following ischemic stroke

### 6.1. Valproic acid

Valproic acid (VPA), a classical anticonvulsive and antimanic agent, has been shown to promote neuronal regeneration in primary rat cortical neurons following hypoxia-reoxygenation via increased BDNF expression and activation 149. Furthermore, VPA enhanced neuronal differentiation of NSCs through the PI3K/Akt/mTOR signaling pathway 150, and increased the expression of miR-210-3p, miR-29a-5p, and miR-674-5p 151. Studies have shown that VPA inhibited glycogen synthase kinase-3β (GSK3β) 152, and reduced GSK3β expression in response to VPA increased neuronal growth in the adult dentate gyrus (DG) in a rodent mood disorder model 153. Furthermore, a study showed that inhibition of GSK3β increased pituitary adenylate cyclase-activating polypeptide (PACAP)-induced neuritogenesis through activation of Rap1 in a Cav-1-dependent manner in PC12 cells 154. These findings indicated that valproate may modulate multiple signaling targets related associated with Cav-1 to improve neuroplasticity after cerebral ischemic injury, suggesting the potential for further translational research.

### 6.2. Resveratrol

Resveratrol (3,5,4'-trihydroxy-trans-stilbene; RSV) is a phenol and phytoalexin found in grapes, red berries, and nuts 155. RSV has been shown to enhance hippocampal plasticity through activation of the histone deacetylase enzyme sirtuin 1 (SIRT1) and AMP-activated kinase (AMPK), resulting in neurite outgrowth 156. Recent studies have shown that RSV exerted protective effects against several neurological diseases including epilepsy 157, Alzheimer's disease 158, and age-related degeneration 159. Furthermore, a study showed that RSV increased proliferation of NSCs and neurite outgrowth via the Shh signaling pathway following OGD/reoxygenation injury *in vitro* 89. Neurological recovery induced by RSV was attributed to angioneurogenesis rather than neuroprotection 160. Resveratrol may increase phosphorylation of Cav-1, c-Src, and eNOS in endothelial cells 161, and Cav-1 was shown to enhance RSV transport in HepG2 cells^162^. Another study showed that RSV promoted neovascularization and Cav-1 interaction with angiogenic molecules in hypercholesterolemic rats 163. Peng et al. showed the effects of RSV on high glucose diet-induced vascular hyperpermeability through Cav-1/eNOS regulation 164. These findings suggest that RSV may be a good drug candidate to induce neuroplasticity after stroke.

### 6.3. Sildenafil

Sildenafil (Viagra®) is an inhibitor of phosphodiesterase-5, and is used to treat erectile dysfunction 165. Studies have shown that sildenafil may reduce neuronal apoptosis, and increase angiogenesis and cerebral blood flow, resulting in functional recovery after ischemic stroke 166. Studies showed that sildenafil enhanced neuroplasticity through activation of the PI3-K/Akt/GSK-3 167 and MAPK/ERK 168 signaling pathways in NSCs. Moreover, increased neuronal growth induced by sildenafil was also observed in NSCs in the SVZ 169 and the DG 170 after ischemic stroke. Studies have shown that sildenafil may restore Cav-1 expression to protect cavernous tissue following pelvic nerve injury 171, and may increase the expression of Cav-1 in dorsal nerve tissues of aged rats 172. Further investigation of the neuroprotective effects of sildenafil in stroke is needed.

### 6.4. Other agents

Other agents may also target Cav-1 to enhance neuronal growth and neuroplasticity following ischemic stroke. Tanshinone I was shown to promote neuronal growth by increasing the expression of Wnt-3, p-GSK-3β, and β-catenin in mouse DG 173. Another study showed that tanshinone IIA promoted neuronal differentiation via activation of Cav-1 and the MAPK42/44/BDNF/NGF signaling pathway 174. Xu et al. demonstrated that a recombinant human IgM, rHIgM12, promoted axonal outgrowth through binding to MLR domains 175. The hypoglycemic drug rosiglitazone was reported to up-regulate Cav-1 expression and activate Src, EGFR, and the MAPK/ERK pathways 176, and to exert neuroprotective effects against acute brain injury 177.

## 7. Future perspectives

The effects of neuronal growth and neuroplasticity following stroke have received increased attention in recent years. Expansion of current studies might result in novel therapeutic options to restore neurological functions in patients that suffered strokes. Recent studies have shown that Cav-1 and MLRs are involved in regulation of neuronal growth and neuroplasticity after ischemic stroke. Many of these studies suggested that Cav-1 may be a promising molecular target to improve neuroplasticity. This review discussed the receptors, signaling pathways, genes, and treatment agents involved in Cav-1-mediated neuroprotection. Although more studies are needed to evaluate the efficacy and safety of the agents discussed in this manuscript, these agents should be evaluated further as treatment options to improve neuroplasticity following ischemic stroke. Further characterization of the roles of Cav-1 and MLRs in neuronal growth and neuroplasticity may provide for novel Cav-1/MLR-based therapies to treat cerebral ischemia. Additional studies are needed to determine whether findings in cells and rats can be translated to clinical applications in humans.

## Figures and Tables

**Fig 1:**
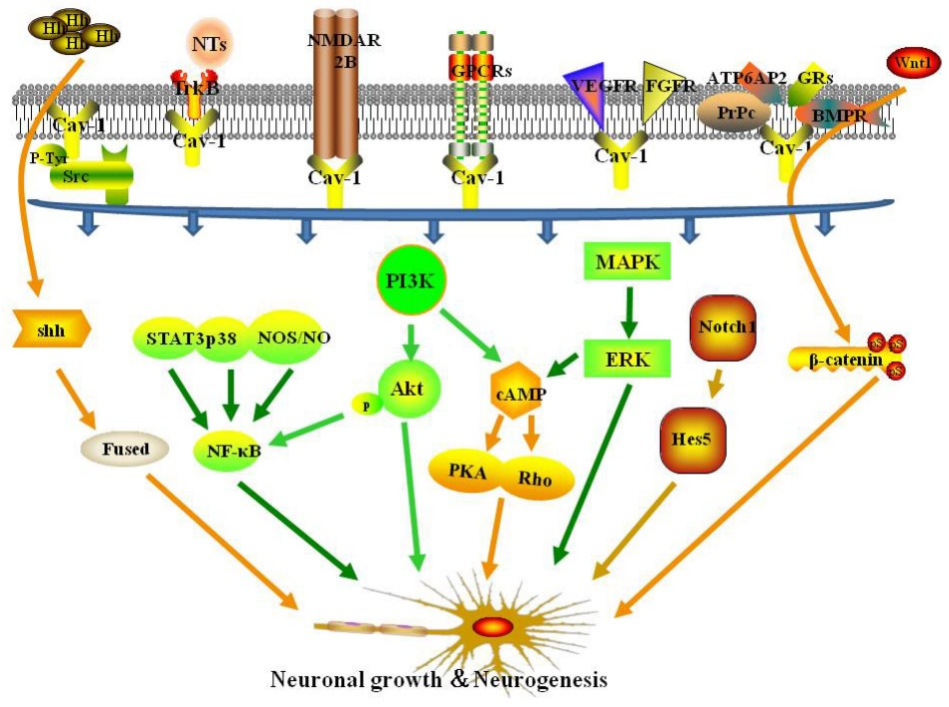
Cav-1- and MLR-associated receptors and signaling pathways in neuronal growth and neuroplasticity following ischemic stroke.

**Fig 2:**
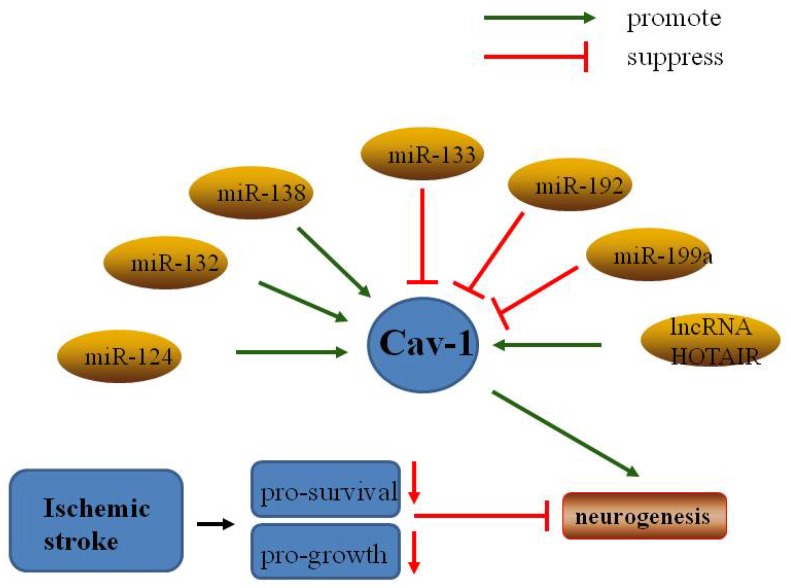
Non-coding RNAs regulate Cav-1 in neuronal growth and neuroplasticity after ischemic stroke.

**Fig 3:**
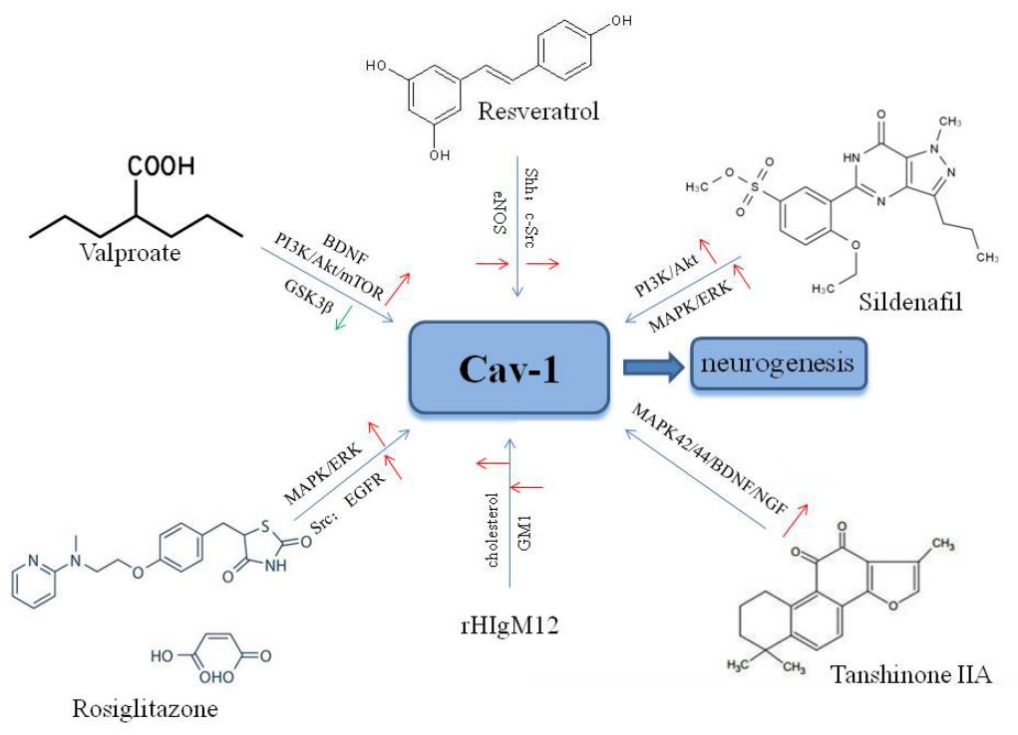
Agents targeting Cav-1 for neuronal growth and neuroplasticity after ischemic stroke.

## References

[1] Tang X, Zhong W, Tu Q (2014). NADPH oxidase mediates the expression of MMP-9 in cerebral tissue after ischemia-reperfusion damage.[J]. Neurol Res.

[2] Demaerschalk B M, Kleindorfer D O, Adeoye O M (2016). Scientific Rationale for the Inclusion and Exclusion Criteria for Intravenous Alteplase in Acute Ischemic Stroke: A Statement for Healthcare Professionals From the American Heart Association/American Stroke Association.[J]. Stroke.

[3] Zhang C, Wu H, Zhu X (2011). Role of transcription factors in neurogenesis after cerebral ischemia.[J]. Rev Neurosci.

[4] Nyberg F (2014). Structural plasticity of the brain to psychostimulant use.[J]. Neuropharmacology.

[5] Havranek T, Zatkova M, Lestanova Z (2015). Intracerebroventricular oxytocin administration in rats enhances object recognition and increases expression of neurotrophins, microtubule-associated protein 2, and synapsin I.[J]. J Neurosci Res.

[6] Koh S H, Park H H (2017). Neurogenesis in Stroke Recovery.[J]. Transl Stroke Res.

[7] Yu T S, Washington P M, Kernie S G (2016). Injury-Induced Neurogenesis: Mechanisms and Relevance.[J]. Neuroscientist.

[8] Pereira P A, Millner T, Vilela M (2016). Nerve growth factor-induced plasticity in medial prefrontal cortex interneurons of aged Wistar rats.[J]. Exp Gerontol.

[9] Petrov A M, Zefirov A L (2013). [Cholesterol and lipid rafts in the biological membranes. Role in the release, reception and ion channel functions].[J]. Usp Fiziol Nauk.

[10] Agarwal S R, Yang P C, Rice M (2014). Role of membrane microdomains in compartmentation of cAMP signaling.[J]. PLoS One.

[11] Busija A R, Patel H H, Insel P A (2017). Caveolins and cavins in the trafficking, maturation, and degradation of caveolae: implications for cell physiology.[J]. Am J Physiol Cell Physiol.

[12] Yin H, Liu T, Zhang Y (2016). Caveolin proteins: a molecular insight into disease.[J]. Front Med.

[13] Ikezu T, Ueda H, Trapp B D (1998). Affinity-purification and characterization of caveolins from the brain: differential expression of caveolin-1, -2, and -3 in brain endothelial and astroglial cell types.[J]. Brain Res.

[14] Razani B, Wang X B, Engelman J A (2002). Caveolin-2-deficient mice show evidence of severe pulmonary dysfunction without disruption of caveolae.[J]. Mol Cell Biol.

[15] Egawa J, Pearn M L, Lemkuil B P (2016). Membrane lipid rafts and neurobiology: age-related changes in membrane lipids and loss of neuronal function.[J]. J Physiol.

[16] Sowa G (2012). Caveolae, caveolins, cavins, and endothelial cell function: new insights.[J]. Front Physiol.

[17] Egawa J, Pearn M L, Lemkuil B P (2016). Membrane lipid rafts and neurobiology: age-related changes in membrane lipids and loss of neuronal function.[J]. J Physiol.

[18] Uittenbogaard A, Smart E J (2000). Palmitoylation of caveolin-1 is required for cholesterol binding, chaperone complex formation, and rapid transport of cholesterol to caveolae.[J]. J Biol Chem.

[19] Shikanai M, Nishimura Y V, Sakurai M (2018). Caveolin-1 Promotes Early Neuronal Maturation via Caveolae-Independent Trafficking of N-Cadherin and L1.[J]. iScience.

[20] Egawa J, Zemljic-Harpf A, Mandyam C D (2017). Neuron-Targeted Caveolin-1 Promotes Ultrastructural and Functional Hippocampal Synaptic Plasticity.[J]. Cereb Cortex.

[21] Head B P, Patel H H, Insel P A (2014). Interaction of membrane/lipid rafts with the cytoskeleton: impact on signaling and function: membrane/lipid rafts, mediators of cytoskeletal arrangement and cell signaling.[J]. Biochim Biophys Acta.

[22] Head B P, Hu Y, Finley J C (2011). Neuron-targeted caveolin-1 protein enhances signaling and promotes arborization of primary neurons.[J]. J Biol Chem.

[23] Egawa J, Schilling J M, Cui W (2017). Neuron-specific caveolin-1 overexpression improves motor function and preserves memory in mice subjected to brain trauma.[J]. FASEB J.

[24] Sawada A, Wang S, Jian M (2019). Neuron-targeted caveolin-1 improves neuromuscular function and extends survival in SOD1(G93A) mice.[J]. FASEB J.

[25] Kumar A, Jaggi A S, Singh N (2015). Pharmacology of Src family kinases and therapeutic implications of their modulators.[J]. Fundam Clin Pharmacol.

[26] Schlegel A, Volonte D, Engelman J A (1998). Crowded little caves: structure and function of caveolae.[J]. Cell Signal.

[27] Radel C, Rizzo V (2005). Integrin mechanotransduction stimulates caveolin-1 phosphorylation and recruitment of Csk to mediate actin reorganization.[J]. Am J Physiol Heart Circ Physiol.

[28] Grande-Garcia A, Echarri A, de Rooij J (2007). Caveolin-1 regulates cell polarization and directional migration through Src kinase and Rho GTPases.[J]. J Cell Biol.

[29] Wang Y, Song Y, Che X (2018). Caveolin1 enhances RANKLinduced gastric cancer cell migration.[J].

[30] Groveman B R, Feng S, Fang X Q (2012). The regulation of N-methyl-D-aspartate receptors by Src kinase.[J]. FEBS J.

[31] Head B P, Patel H H, Tsutsumi Y M (2008). Caveolin-1 expression is essential for N-methyl-D-aspartate receptor-mediated Src and extracellular signal-regulated kinase 1/2 activation and protection of primary neurons from ischemic cell death.[J]. FASEB J.

[32] Castren E (2014). Neurotrophins and psychiatric disorders.[J]. Handb Exp Pharmacol.

[33] Lawn S, Krishna N, Pisklakova A (2015). Neurotrophin signaling via TrkB and TrkC receptors promotes the growth of brain tumor-initiating cells.[J]. J Biol Chem.

[34] Qi D, Ouyang C, Wang Y (2014). HO-1 attenuates hippocampal neurons injury via the activation of BDNF-TrkB-PI3K/Akt signaling pathway in stroke.[J]. Brain Res.

[35] Yang Y, Ma Z, Hu W (2016). Caveolin-1/-3: therapeutic targets for myocardial ischemia/reperfusion injury.[J]. Basic Res Cardiol.

[36] Bilderback T R, Gazula V R, Lisanti M P (1999). Caveolin interacts with Trk A and p75(NTR) and regulates neurotrophin signaling pathways.[J]. J Biol Chem.

[37] Hansen K B, Yi F, Perszyk R E (2017). NMDA Receptors in the Central Nervous System.[J]. Methods Mol Biol.

[38] Yang J X, Hua L, Li Y Q (2015). Caveolin-1 in the anterior cingulate cortex modulates chronic neuropathic pain via regulation of NMDA receptor 2B subunit.[J]. J Neurosci.

[39] Eichel K, von Zastrow M (2018). Subcellular Organization of GPCR Signaling.[J]. Trends Pharmacol Sci.

[40] Bockaert J, Perroy J, Becamel C (2010). GPCR interacting proteins (GIPs) in the nervous system: Roles in physiology and pathologies.[J]. Annu Rev Pharmacol Toxicol.

[41] Head B P, Patel H H, Roth D M (2005). G-protein-coupled receptor signaling components localize in both sarcolemmal and intracellular caveolin-3-associated microdomains in adult cardiac myocytes.[J]. J Biol Chem.

[42] Oh P, Schnitzer J E (2001). Segregation of heterotrimeric G proteins in cell surface microdomains. G(q) binds caveolin to concentrate in caveolae, whereas G(i) and G(s) target lipid rafts by default.[J]. Mol Biol Cell.

[43] Mermelstein P G (2009). Membrane-localised oestrogen receptor alpha and beta influence neuronal activity through activation of metabotropic glutamate receptors.[J]. J Neuroendocrinol.

[44] Sun Y, Jin K, Xie L (2003). VEGF-induced neuroprotection, neurogenesis, and angiogenesis after focal cerebral ischemia.[J]. J Clin Invest.

[45] Tahir S A, Park S, Thompson T C (2009). Caveolin-1 regulates VEGF-stimulated angiogenic activities in prostate cancer and endothelial cells.[J]. Cancer Biol Ther.

[46] Zhao Y, Pang Q, Liu M (2017). Treadmill Exercise Promotes Neurogenesis in Ischemic Rat Brains via Caveolin-1/VEGF Signaling Pathways.[J]. Neurochem Res.

[47] Pang Q, Zhang H, Chen Z (2017). Role of caveolin-1/vascular endothelial growth factor pathway in basic fibroblast growth factor-induced angiogenesis and neurogenesis after treadmill training following focal cerebral ischemia in rats.[J]. Brain Res.

[48] Feng L, Liao W X, Luo Q (2012). Caveolin-1 orchestrates fibroblast growth factor 2 signaling control of angiogenesis in placental artery endothelial cell caveolae.[J]. J Cell Physiol.

[49] Mouillet-Richard S, Ermonval M, Chebassier C (2000). Signal transduction through prion protein.[J]. Science.

[50] Taylor D R, Hooper N M (2006). The prion protein and lipid rafts.[J]. Mol Membr Biol.

[51] Pantera B, Bini C, Cirri P (2009). PrPc activation induces neurite outgrowth and differentiation in PC12 cells: role for caveolin-1 in the signal transduction pathway.[J]. J Neurochem.

[52] Nohe A, Keating E, Underhill T M (2005). Dynamics and interaction of caveolin-1 isoforms with BMP-receptors.[J]. J Cell Sci.

[53] Wertz J W, Bauer P M (2008). Caveolin-1 regulates BMPRII localization and signaling in vascular smooth muscle cells.[J]. Biochem Biophys Res Commun.

[54] Du J, Chen X, Liang X (2011). Integrin activation and internalization on soft ECM as a mechanism of induction of stem cell differentiation by ECM elasticity.[J]. Proc Natl Acad Sci U S A.

[55] Makdissy N, Haddad K, AlBacha J D (2018). Essential role of ATP6AP2 enrichment in caveolae/lipid raft microdomains for the induction of neuronal differentiation of stem cells.[J]. Stem Cell Res Ther.

[56] Samarasinghe R A, Di Maio R, Volonte D (2011). Nongenomic glucocorticoid receptor action regulates gap junction intercellular communication and neural progenitor cell proliferation.[J]. Proc Natl Acad Sci U S A.

[57] Cui W, Ren Y, Wang S (2017). The role of caveolin-1 in morphine-induced structural plasticity in primary cultured mouse cerebral cortical neurons.[J]. Neurosci Lett.

[58] Koh S H, Lo E H (2015). The Role of the PI3K Pathway in the Regeneration of the Damaged Brain by Neural Stem Cells after Cerebral Infarction.[J]. J Clin Neurol.

[59] Gao G S, Li Y, Zhai H (2017). Humanin analogue, S14G-humanin, has neuroprotective effects against oxygen glucose deprivation/reoxygenation by reactivating Jak2/Stat3 signaling through the PI3K/AKT pathway.[J]. Exp Ther Med.

[60] Jin X F, Wang S, Shen M (2017). Effects of rehabilitation training on apoptosis of nerve cells and the recovery of neural and motor functions in rats with ischemic stroke through the PI3K/Akt and Nrf2/ARE signaling pathways.[J]. Brain Res Bull.

[61] Yang H, Guan L, Li S (2016). Mechanosensitive caveolin-1 activation-induced PI3K/Akt/mTOR signaling pathway promotes breast cancer motility, invadopodia formation and metastasis in vivo.[J]. Oncotarget.

[62] Baker N, Sohn J, Tuan R S (2015). Promotion of human mesenchymal stem cell osteogenesis by PI3-kinase/Akt signaling, and the influence of caveolin-1/cholesterol homeostasis.[J]. Stem Cell Res Ther.

[63] Penumathsa S V, Thirunavukkarasu M, Samuel S M (2009). Niacin bound chromium treatment induces myocardial Glut-4 translocation and caveolar interaction via Akt, AMPK and eNOS phosphorylation in streptozotocin induced diabetic rats after ischemia-reperfusion injury.[J]. Biochim Biophys Acta.

[64] Umesalma S, Houwen F K, Baumbach G L (2016). Roles of Caveolin-1 in Angiotensin II-Induced Hypertrophy and Inward Remodeling of Cerebral Pial Arterioles.[J]. Hypertension.

[65] Bauer P M, Yu J, Chen Y (2005). Endothelial-specific expression of caveolin-1 impairs microvascular permeability and angiogenesis.[J]. Proc Natl Acad Sci U S A.

[66] Li Y, Luo J, Lau W M (2011). Caveolin-1 plays a crucial role in inhibiting neuronal differentiation of neural stem/progenitor cells via VEGF signaling-dependent pathway.[J]. PLoS One.

[67] Li Q, Chen M, Liu H (2014). The dual role of ERK signaling in the apoptosis of neurons.[J]. Front Biosci (Landmark Ed).

[68] Nonami A, Taketomi T, Kimura A (2005). The Sprouty-related protein, Spred-1, localizes in a lipid raft/caveola and inhibits ERK activation in collaboration with caveolin-1.[J]. Genes Cells.

[69] Haines P, Samuel G H, Cohen H (2011). Caveolin-1 is a negative regulator of MMP-1 gene expression in human dermal fibroblasts via inhibition of Erk1/2/Ets1 signaling pathway.[J]. J Dermatol Sci.

[70] Kwon H, Jeong K, Hwang E M (2011). A novel domain of caveolin-2 that controls nuclear targeting: regulation of insulin-specific ERK activation and nuclear translocation by caveolin-2.[J]. J Cell Mol Med.

[71] Iwakura Y, Zheng Y, Sibilia M (2011). Qualitative and quantitative re-evaluation of epidermal growth factor-ErbB1 action on developing midbrain dopaminergic neurons in vivo and in vitro: target-derived neurotrophic signaling (Part 1).[J]. J Neurochem.

[72] Xu L, Wang L, Wen Z (2016). Caveolin-1 is a checkpoint regulator in hypoxia-induced astrocyte apoptosis via Ras/Raf/ERK pathway.[J]. Am J Physiol Cell Physiol.

[73] Sonveaux P, Martinive P, DeWever J (2004). Caveolin-1 expression is critical for vascular endothelial growth factor-induced ischemic hindlimb collateralization and nitric oxide-mediated angiogenesis.[J]. Circ Res.

[74] Li B, Jia S, Yue T (2017). Biphasic Regulation of Caveolin-1 Gene Expression by Fluoxetine in Astrocytes: Opposite Effects of PI3K/AKT and MAPK/ERK Signaling Pathways on c-fos.[J]. Front Cell Neurosci.

[75] Sarnico I, Lanzillotta A, Benarese M (2009). NF-kappaB dimers in the regulation of neuronal survival.[J]. Int Rev Neurobiol.

[76] Aloor R, Zhang C, Bandyopadhyay M (2015). Impact of nuclear factor-kappaB on restoration of neuron growth and differentiation in hippocampus of degenerative brain.[J]. J Neurosci Res.

[77] Crampton S J, O'Keeffe G W (2013). NF-kappaB: emerging roles in hippocampal development and function.[J]. Int J Biochem Cell Biol.

[78] Zhu X, Zhang Y, Li Q (2016). beta-Carotene Induces Apoptosis in Human Esophageal Squamous Cell Carcinoma Cell Lines via the Cav-1/AKT/NF-kappaB Signaling Pathway.[J]. J Biochem Mol Toxicol.

[79] Yuan K, Huang C, Fox J (2011). Elevated inflammatory response in caveolin-1-deficient mice with Pseudomonas aeruginosa infection is mediated by STAT3 protein and nuclear factor kappaB (NF-kappaB).[J]. J Biol Chem.

[80] Lv X J, Li Y Y, Zhang Y J (2010). Over-expression of caveolin-1 aggravate LPS-induced inflammatory response in AT-1 cells via up-regulation of cPLA2/p38 MAPK.[J]. Inflamm Res.

[81] Garrean S, Gao X P, Brovkovych V (2006). Caveolin-1 regulates NF-kappaB activation and lung inflammatory response to sepsis induced by lipopolysaccharide.[J]. J Immunol.

[82] Patel S S, Tomar S, Sharma D (2017). Targeting sonic hedgehog signaling in neurological disorders.[J]. Neurosci Biobehav Rev.

[83] Berretta A, Gowing E K, Jasoni C L (2016). Sonic hedgehog stimulates neurite outgrowth in a mechanical stretch model of reactive-astrogliosis.[J]. Sci Rep.

[84] Hung H C, Hsiao Y H, Gean P W (2014). Learning induces sonic hedgehog signaling in the amygdala which promotes neurogenesis and long-term memory formation.[J].

[85] Hung H C, Hsiao Y H, Gean P W (2015). Sonic hedgehog signaling regulates amygdalar neurogenesis and extinction of fear memory.[J]. Eur Neuropsychopharmacol.

[86] Bambakidis N C, Onwuzulike K (2012). Sonic Hedgehog signaling and potential therapeutic indications.[J]. Vitam Horm.

[87] Zhang L, Chopp M, Meier D H (2013). Sonic hedgehog signaling pathway mediates cerebrolysin-improved neurological function after stroke.[J]. Stroke.

[88] Zhang Y, Zhang X, Cui L (2017). Salvianolic Acids for Injection (SAFI) promotes functional recovery and neurogenesis via sonic hedgehog pathway after stroke in mice.[J]. Neurochem Int.

[89] Tang F, Guo S, Liao H (2017). Resveratrol Enhances Neurite Outgrowth and Synaptogenesis Via Sonic Hedgehog Signaling Following Oxygen-Glucose Deprivation/Reoxygenation Injury.[J]. Cell Physiol Biochem.

[90] Yue S, Tang L Y, Tang Y (2014). Requirement of Smurf-mediated endocytosis of Patched1 in sonic hedgehog signal reception.[J].

[91] Tsai M T, Cheng C J, Lin Y C (2009). Isolation and characterization of a secreted, cell-surface glycoprotein SCUBE2 from humans.[J]. Biochem J.

[92] Mao H, Diehl A M, Li Y X (2009). Sonic hedgehog ligand partners with caveolin-1 for intracellular transport.[J]. Lab Invest.

[93] Lee D (2015). Global and local missions of cAMP signaling in neural plasticity, learning, and memory.[J]. Front Pharmacol.

[94] Ghiglieri V, Napolitano F, Pelosi B (2015). Rhes influences striatal cAMP/PKA-dependent signaling and synaptic plasticity in a gender-sensitive fashion.[J]. Sci Rep.

[95] Koinuma S, Takeuchi K, Wada N (2017). cAMP-induced activation of protein kinase A and p190B RhoGAP mediates down-regulation of TC10 activity at the plasma membrane and neurite outgrowth.[J]. Genes Cells.

[96] Kuo A, Lee M Y, Yang K (2018). Caveolin-1 regulates lipid droplet metabolism in endothelial cells via autocrine prostacyclin-stimulated, cAMP-mediated lipolysis.[J]. J Biol Chem.

[97] Jasmin J F, Rengo G, Lymperopoulos A (2011). Caveolin-1 deficiency exacerbates cardiac dysfunction and reduces survival in mice with myocardial infarction.[J]. Am J Physiol Heart Circ Physiol.

[98] Leal G, Comprido D, Duarte C B (2014). BDNF-induced local protein synthesis and synaptic plasticity.[J].

[99] Scholzke M N, Schwaninger M (2007). Transcriptional regulation of neurogenesis: potential mechanisms in cerebral ischemia.[J]. J Mol Med (Berl).

[100] Engler A, Zhang R, Taylor V (2018). Notch and Neurogenesis.[J]. Adv Exp Med Biol.

[101] Sun F, Mao X, Xie L (2013). Notch1 signaling modulates neuronal progenitor activity in the subventricular zone in response to aging and focal ischemia.[J]. Aging Cell.

[102] Zou W, Ma X, Hua W (2015). Caveolin-1 mediates chemoresistance in cisplatin-resistant ovarian cancer cells by targeting apoptosis through the Notch-1/Akt/NF-kappaB pathway.[J]. Oncol Rep.

[103] Campos L S, Decker L, Taylor V (2006). Notch, epidermal growth factor receptor, and beta1-integrin pathways are coordinated in neural stem cells.[J]. J Biol Chem.

[104] Wang S, Kan Q, Sun Y (2013). Caveolin-1 regulates neural differentiation of rat bone mesenchymal stem cells into neurons by modulating Notch signaling.[J]. Int J Dev Neurosci.

[105] Li Y, Lau W M, So K F (2011). Caveolin-1 promote astroglial differentiation of neural stem/progenitor cells through modulating Notch1/NICD and Hes1 expressions.[J]. Biochem Biophys Res Commun.

[106] Mo S, Wang L, Li Q (2010). Caveolin-1 regulates dorsoventral patterning through direct interaction with beta-catenin in zebrafish.[J]. Dev Biol.

[107] Sotgia F, Williams T M, Cohen A W (2005). Caveolin-1-deficient mice have an increased mammary stem cell population with upregulation of Wnt/beta-catenin signaling.[J]. Cell Cycle.

[108] Li Y, Lau W M, So K F (2011). Caveolin-1 inhibits oligodendroglial differentiation of neural stem/progenitor cells through modulating beta-catenin expression.[J]. Neurochem Int.

[109] Saugstad J A (2015). Non-Coding RNAs in Stroke and Neuroprotection.[J]. Front Neurol.

[110] Hammond S M, Bernstein E, Beach D (2000). An RNA-directed nuclease mediates post-transcriptional gene silencing in Drosophila cells.[J]. Nature.

[111] Zhang Y, Guo J (2012). MicroRNA and cerebral ischemia.[J]. Zhongguo Yi Xue Ke Xue Yuan Xue Bao.

[112] Liu X S, Chopp M, Zhang R L (2013). MicroRNAs in cerebral ischemia-induced neurogenesis.[J]. J Neuropathol Exp Neurol.

[113] Qiu J, Zhou X Y, Zhou X G (2013). Neuroprotective effects of microRNA-210 against oxygen-glucose deprivation through inhibition of apoptosis in PC12 cells.[J]. Mol Med Rep.

[114] Zeng L L, He X S, Liu J R (2016). Lentivirus-Mediated Overexpression of MicroRNA-210 Improves Long-Term Outcomes after Focal Cerebral Ischemia in Mice.[J]. CNS Neurosci Ther.

[115] Liu X S, Chopp M, Wang X L (2013). MicroRNA-17-92 cluster mediates the proliferation and survival of neural progenitor cells after stroke.[J]. J Biol Chem.

[116] Cheng L C, Pastrana E, Tavazoie M (2009). miR-124 regulates adult neurogenesis in the subventricular zone stem cell niche.[J]. Nat Neurosci.

[117] Maiorano N A, Mallamaci A (2010). The pro-differentiating role of miR-124: indicating the road to become a neuron.[J]. RNA Biol.

[118] Hamzei T S, Kho W, Aswendt M (2016). Dynamic Modulation of Microglia/Macrophage Polarization by miR-124 after Focal Cerebral Ischemia.[J]. J Neuroimmune Pharmacol.

[119] Wu Y, Zhong A, Zheng H (2015). Expression of Flotilin-2 and Acrosome Biogenesis Are Regulated by MiR-124 during Spermatogenesis.[J]. PLoS One.

[120] Yang S, Liu X, Li X (2013). MicroRNA-124 reduces caveolar density by targeting caveolin-1 in porcine kidney epithelial PK15 cells.[J]. Mol Cell Biochem.

[121] Kang Q, Xiang Y, Li D (2017). MiR-124-3p attenuates hyperphosphorylation of Tau protein-induced apoptosis via caveolin-1-PI3K/Akt/GSK3beta pathway in N2a/APP695swe cells.[J]. Oncotarget.

[122] Liu X S, Chopp M, Zhang R L (2011). MicroRNA profiling in subventricular zone after stroke: MiR-124a regulates proliferation of neural progenitor cells through Notch signaling pathway.[J]. PLoS One.

[123] Xu W H, Yao X Y, Yu H J (2012). Downregulation of miR-199a may play a role in 3-nitropropionic acid induced ischemic tolerance in rat brain.[J]. Brain Res.

[124] Lv H R (2017). lncRNA-Map2k4 sequesters miR-199a to promote FGF1 expression and spinal cord neuron growth.[J]. Biochem Biophys Res Commun.

[125] Bao N, Fang B, Lv H (2018). Upregulation of miR-199a-5p Protects Spinal Cord Against Ischemia/Reperfusion-Induced Injury via Downregulation of ECE1 in Rat.[J]. Cell Mol Neurobiol.

[126] Aranda J F, Canfran-Duque A, Goedeke L (2015). The miR-199-dynamin regulatory axis controls receptor-mediated endocytosis.[J]. J Cell Sci.

[127] Shi X E, Li Y F, Jia L (2014). MicroRNA-199a-5p affects porcine preadipocyte proliferation and differentiation.[J]. Int J Mol Sci.

[128] Yu H, Wu M, Zhao P (2015). Neuroprotective effects of viral overexpression of microRNA-22 in rat and cell models of cerebral ischemia-reperfusion injury.[J]. J Cell Biochem.

[129] Chen Z, Qi Y, Gao C (2015). Cardiac myocyte-protective effect of microRNA-22 during ischemia and reperfusion through disrupting the caveolin-3/eNOS signaling.[J]. Int J Clin Exp Pathol.

[130] Pathania M, Torres-Reveron J, Yan L (2012). miR-132 enhances dendritic morphogenesis, spine density, synaptic integration, and survival of newborn olfactory bulb neurons.[J]. PLoS One.

[131] Gu Y, Cai R, Zhang C (2018). miR-132-3p boosts caveolae-mediated transcellular transport in glioma endothelial cells by targeting PTEN/PI3K/PKB/Src/Cav-1 signaling pathway.[J]. FASEB J.

[132] Xin H, Li Y, Buller B (2012). Exosome-mediated transfer of miR-133b from multipotent mesenchymal stromal cells to neural cells contributes to neurite outgrowth.[J]. Stem Cells.

[133] Nohata N, Hanazawa T, Kikkawa N (2011). Caveolin-1 mediates tumor cell migration and invasion and its regulation by miR-133a in head and neck squamous cell carcinoma.[J]. Int J Oncol.

[134] Tang X J, Yang M H, Cao G (2016). Protective effect of microRNA-138 against cerebral ischemia/reperfusion injury in rats.[J]. Exp Ther Med.

[135] Gong H, Song L, Lin C (2013). Downregulation of miR-138 sustains NF-kappaB activation and promotes lipid raft formation in esophageal squamous cell carcinoma.[J]. Clin Cancer Res.

[136] Li S, Jin Z, Lu X (2017). MicroRNA-192 suppresses cell proliferation and induces apoptosis in human rheumatoid arthritis fibroblast-like synoviocytes by downregulating caveolin 1.[J]. Mol Cell Biochem.

[137] Ng S Y, Lin L, Soh B S (2013). Long noncoding RNAs in development and disease of the central nervous system.[J]. Trends Genet.

[138] Dharap A, Nakka V P, Vemuganti R (2012). Effect of focal ischemia on long noncoding RNAs.[J]. Stroke.

[139] Ayana R, Singh S, Pati S (2017). Decoding Crucial LncRNAs Implicated in Neurogenesis and Neurological Disorders.[J]. Stem Cells Dev.

[140] Pandey G K, Mitra S, Subhash S (2014). The risk-associated long noncoding RNA NBAT-1 controls neuroblastoma progression by regulating cell proliferation and neuronal differentiation.[J]. Cancer Cell.

[141] Peschansky V J, Pastori C, Zeier Z (2016). The long non-coding RNA FMR4 promotes proliferation of human neural precursor cells and epigenetic regulation of gene expression in trans.[J]. Mol Cell Neurosci.

[142] Ramos A D, Andersen R E, Liu S J (2015). The long noncoding RNA Pnky regulates neuronal differentiation of embryonic and postnatal neural stem cells.[J]. Cell Stem Cell.

[143] Yue Y, Zhang W, Liu C (2015). [Long non-coding RNA Gm15577 is involved in mouse cerebellar neurogenesis].[J]. Zhonghua Bing Li Xue Za Zhi.

[144] Josipovic I, Fork C, Preussner J (2016). PAFAH1B1 and the lncRNA NONHSAT073641 maintain an angiogenic phenotype in human endothelial cells.[J]. Acta Physiol (Oxf).

[145] Chen L, Feng P, Zhu X (2016). Long non-coding RNA Malat1 promotes neurite outgrowth through activation of ERK/MAPK signalling pathway in N2a cells.[J]. J Cell Mol Med.

[146] Rani N, Nowakowski T J, Zhou H (2016). A Primate lncRNA Mediates Notch Signaling during Neuronal Development by Sequestering miRNA.[J]. Neuron.

[147] Yang L, Lu Z N (2016). Long non-coding RNA HOTAIR promotes ischemic infarct induced by hypoxia through up-regulating the expression of NOX2.[J]. Biochem Biophys Res Commun.

[148] Liu W, Yin N C, Liu H (2018). Cav-1 promote lung cancer cell proliferation and invasion through lncRNA HOTAIR.[J]. Gene.

[149] Hasan M R, Kim J H, Kim Y J (2013). Effect of HDAC inhibitors on neuroprotection and neurite outgrowth in primary rat cortical neurons following ischemic insult.[J]. Neurochem Res.

[150] Zhang X, He X, Li Q (2017). PI3K/AKT/mTOR Signaling Mediates Valproic Acid-Induced Neuronal Differentiation of Neural Stem Cells through Epigenetic Modifications.[J]. Stem Cell Reports.

[151] He H, Li W, Peng M (2018). MicroRNA expression profiles of neural stem cells following valproate inducement.[J]. J Cell Biochem.

[152] Kao C Y, Hsu Y C, Liu J W (2013). The mood stabilizer valproate activates human FGF1 gene promoter through inhibiting HDAC and GSK-3 activities.[J]. J Neurochem.

[153] Boku S, Nakagawa S, Masuda T (2014). Valproate recovers the inhibitory effect of dexamethasone on the proliferation of the adult dentate gyrus-derived neural precursor cells via GSK-3beta and beta-catenin pathway.[J]. Eur J Pharmacol.

[154] Zhang W, Smith A, Liu J P (2009). GSK3beta modulates PACAP-induced neuritogenesis in PC12 cells by acting downstream of Rap1 in a caveolae-dependent manner.[J]. Cell Signal.

[155] Safahani M, Aligholi H, Noorbakhsh F (2018). Resveratrol promotes the arcuate nucleus architecture remodeling to produce more anorexigenic neurons in high-fat-diet-fed mice.[J]. Nutrition.

[156] Dias G P, Cocks G, do N B M (2016). Resveratrol: A Potential Hippocampal Plasticity Enhancer.[J]. Oxid Med Cell Longev.

[157] Mishra V, Shuai B, Kodali M (2015). Resveratrol Treatment after Status Epilepticus Restrains Neurodegeneration and Abnormal Neurogenesis with Suppression of Oxidative Stress and Inflammation.[J]. Sci Rep.

[158] Wang X, Ma S, Yang B (2018). Resveratrol promotes hUC-MSCs engraftment and neural repair in a mouse model of Alzheimer's disease.[J]. Behav Brain Res.

[159] Kumar V, Pandey A, Jahan S (2016). Differential responses of Trans-Resveratrol on proliferation of neural progenitor cells and aged rat hippocampal neurogenesis.[J]. Sci Rep.

[160] Hermann D M, Zechariah A, Kaltwasser B (2015). Sustained neurological recovery induced by resveratrol is associated with angioneurogenesis rather than neuroprotection after focal cerebral ischemia.[J]. Neurobiol Dis.

[161] Klinge C M, Wickramasinghe N S, Ivanova M M (2008). Resveratrol stimulates nitric oxide production by increasing estrogen receptor alpha-Src-caveolin-1 interaction and phosphorylation in human umbilical vein endothelial cells.[J]. FASEB J.

[162] Yang H L, Chen W Q, Cao X (2009). Caveolin-1 enhances resveratrol-mediated cytotoxicity and transport in a hepatocellular carcinoma model.[J]. J Transl Med.

[163] Penumathsa S V, Thirunavukkarasu M, Zhan L (2008). Resveratrol enhances GLUT-4 translocation to the caveolar lipid raft fractions through AMPK/Akt/eNOS signalling pathway in diabetic myocardium.[J]. J Cell Mol Med.

[164] Peng X L, Qu W, Wang L Z (2014). Resveratrol ameliorates high glucose and high-fat/sucrose diet-induced vascular hyperpermeability involving Cav-1/eNOS regulation.[J]. PLoS One.

[165] Gur S, Kadowitz P J, Serefoglu E C (2012). PDE5 inhibitor treatment options for urologic and non-urologic indications: 2012 update.[J]. Curr Pharm Des.

[166] Olmestig J, Marlet I R, Hainsworth A H (2017). Phosphodiesterase 5 inhibition as a therapeutic target for ischemic stroke: A systematic review of preclinical studies.[J]. Cell Signal.

[167] Wang L, Gang Z Z, Lan Z R (2005). Activation of the PI3-K/Akt pathway mediates cGMP enhanced-neurogenesis in the adult progenitor cells derived from the subventricular zone.[J]. J Cereb Blood Flow Metab.

[168] Santos A I, Carreira B P, Nobre R J (2014). Stimulation of neural stem cell proliferation by inhibition of phosphodiesterase 5.[J]. Stem Cells Int.

[169] Zhang R L, Chopp M, Roberts C (2012). Sildenafil enhances neurogenesis and oligodendrogenesis in ischemic brain of middle-aged mouse.[J]. PLoS One.

[170] Zhang R, Wang Y, Zhang L (2002). Sildenafil (Viagra) induces neurogenesis and promotes functional recovery after stroke in rats.[J]. Stroke.

[171] Becher E F, Toblli J E, Castronuovo C (2009). Expression of caveolin-1 in penile cavernosal tissue in a denervated animal model after treatment with sildenafil citrate.[J]. J Sex Med.

[172] Kovac J R, DeYoung L, Lehmann K J (2014). The effects of combined free radical scavenger and sildenafil therapy on age-associated erectile dysfunction: An animal model.[J]. Urol Ann.

[173] Chen B H, Park J H, Cho J H (2016). Tanshinone I Enhances Neurogenesis in the Mouse Hippocampal Dentate Gyrus via Increasing Wnt-3, Phosphorylated Glycogen Synthase Kinase-3beta and beta-Catenin Immunoreactivities.[J]. Neurochem Res.

[174] Zhao Y, Xu P, Hu S (2015). Tanshinone II A, a multiple target neuroprotectant, promotes caveolae-dependent neuronal differentiation.[J]. Eur J Pharmacol.

[175] Xu X, Warrington A E, Wright B R (2011). A human IgM signals axon outgrowth: coupling lipid raft to microtubules.[J]. J Neurochem.

[176] Tencer L, Burgermeister E, Ebert M P (2008). Rosiglitazone induces caveolin-1 by PPARgamma-dependent and PPRE-independent mechanisms: the role of EGF receptor signaling and its effect on cancer cell drug resistance.[J]. Anticancer Res.

[177] Zhao Y L, Song J N, Ma X D (2016). Rosiglitazone ameliorates diffuse axonal injury by reducing loss of tau and up-regulating caveolin-1 expression.[J]. Neural Regen Res.

